# Characteristics of Tumor Infiltrating Lymphocyte and Circulating Lymphocyte Repertoires in Pancreatic Cancer by the Sequencing of T Cell Receptors

**DOI:** 10.1038/srep13664

**Published:** 2015-09-02

**Authors:** Xueli Bai, Qi Zhang, Song Wu, Xiaoyu Zhang, Mingbang Wang, Fusheng He, Tao Wei, Jiaqi Yang, Yu Lou, Zhiming Cai, Tingbo Liang

**Affiliations:** 1 Department of Hepatobiliary and Pancreatic Surgery, the Second Affiliated Hospital, Zhejiang University School of Medicine, Hangzhou, China; 2 Key Laboratory of Cancer Prevention and Intervention, the Second Affiliated Hospital, Zhejiang University School of Medicine, Hangzhou, China; 3 National-regional Key Technology Engineering Laboratory for Clinical Application of Cancer Genomics, Shenzhen Second People’s Hospital, the First Affiliated Hospital of Shenzhen University, Shenzhen, China; 4 Siteman Cancer Center, Washington University in St. Louis, St. Louis, Missouri, USA; 5 Shenzhen Following Precision Medical Research Institute; 6 Zhejiang University; Collaborative Innovation Center for Cancer Medicine, Guangzhou, China

## Abstract

Pancreatic cancer has a poor prognosis and few effective treatments. The failure of treatment is partially due to the high heterogeneity of cancer cells within the tumor. T cells target and kill cancer cells by the specific recognition of cancer-associated antigens. In this study, T cells from primary tumor and blood of sixteen patients with pancreatic cancer were characterized by deep sequencing. T cells from blood of another eight healthy volunteers were also studied as controls. By analyzing the *complementary determining region 3* (*CDR3*) gene sequence, we found no significant differences in the T cell receptor (TCR) repertoires between patients and healthy controls. Types and length of CDR3 were similar among groups. However, two clusters of patients were identified according to the degree of CDR3 overlap within tumor sample group. In addition, clonotypes with low frequencies were found in significantly higher numbers in primary pancreatic tumors compared to blood samples from patients and healthy controls. This study is the first to characterize the TCR repertoires of pancreatic cancers in both primary tumors and matched blood samples. The results imply that specific types of pancreatic cancer share potentially important immunological characteristics.

Pancreatic cancer is one of the most deadly malignant diseases, with a 5-year survival rate of only 6%^1^. No efficient therapeutic strategies have been developed to date in most cases. Extensive efforts have been made for decades to find specific targets in pancreatic cancer^2^. Immune cells, such as CD8^+^ T cells, can naturally remove cancer cells, which implies that certain subclones specifically recognize cancer cells by specific interactions with the T-cell receptor (TCR) and tumor-associated peptides. These T cell subclones may contribute significantly to the efficacy of anti-cancer immunotherapy including immune checkpoint blockades^3^ and chimeric antigen receptor T cell-based therapeutics^4^.

T cells can be classified into two groups in patients with cancer: namely, tumor-infiltrating T cells and circulating T cells. These two components of the T cell repertoire are associated with each other but have distinct distributions of subclones, as well as different clinical significance^5^. It has been well documented that the number or proportion of tumor infiltrating lymphocytes (TILs) is associated with a better prognosis in various types of cancer^6^. However, cancer cells can find ways to escape the immune response by exhausting or inactivating TILs^7^. It is therefore important to identify the T cell subclones that can kill cancer cells efficiently, and attempt to enhance them by different strategies.

TILs are exposed to the changing tumor microenvironment which is anisotropic in position and changes as tumor progresses^8^. Heterogeneity in cancer has been partially investigated through single cell sequencing of circulating tumor cells^9^. The TCR repertoire, from another perspective, shows extensive heterogeneity in solid cancers, including pancreatic cancer^10^. From the results of high-throughput sequencing, investigators can now analyze the TCR repertoire and identify T cell subclones^11^. The TCR is responsible for T cell specificity, and is composed of two polypeptide chains in either an αβ type or a γδ type^12^. The complementary determining region 3 (CDR3) of the β chain further determines the antigenic specificity of T cells. CDR3 includes variable (V), diversity (D), and joining (J) regions encoded by the corresponding V, D, and J genes. The rearrangement of the V, D, and J regions ensures that CDR3 has a high potential for diversity^13^, and makes it possible for T cells to target any antigens from either endogenous or exogenous sources^14^. Hence, sequencing the TCR repertoire is an accurate method to assess the immune response status of cancer patients.

High-throughput sequencing has been used to define T cell repertoires in blood samples of healthy individuals as well as tumor samples^15^. Most related studies have focused on different tumor sites to determine the heterogeneity or homogeneity of the tumor^16^,^17^. However, no reports to date have been published regarding pancreatic cancer, and few studies have emphasized the correlation between TILs and circulating T cells with regards to their TCR repertoires. In this study, we investigated the T cell repertoire in patients with pancreatic cancer (both blood and tumor samples) as well as those of healthy control individuals using high-throughput sequencing. These results deepen our understanding of immune system responses in patients with pancreatic cancer.

## Results

**Figure 1:**
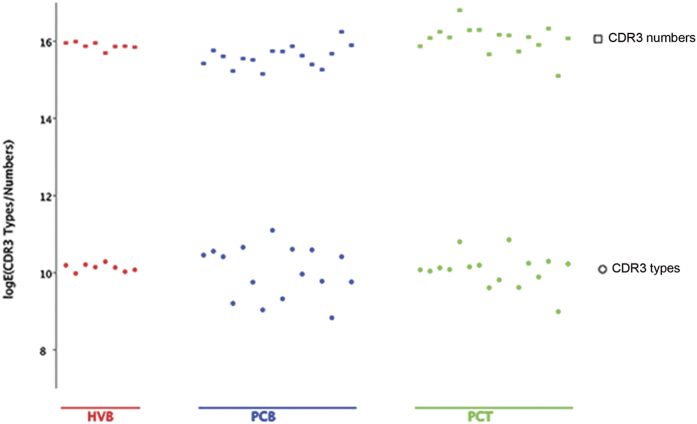
Types and numbers of complementary determining region 3 (CDR3) in each group. Types and numbers of CDR3 were similar among groups; however, levels of diversity seemed higher in tissue and blood samples from patients compared to blood samples from healthy controls.

An Average of 18 (range, 13 to 36) million sequencing reads were acquired from each sample in our cohort. The numbers of clonotypes were 93,925 ± 32,490, 76,093 ± 34,020, and 93,527 ± 9,392 in the pancreatic cancer tissue (PCT), pancreatic cancer blood (PCB), and healthy volunteer blood (HVB) groups, respectively (Table S1). The numbers of unique peptides detected were 69,467 ± 27,035, 62,162 ± 30,135, and 70,265 ± 6,937 in the PCT, PCB, and HVB groups, respectively. The total numbers and types of CDR3 were at the same level in all groups, although samples from the patients showed a higher level of diversity within the two groups (Fig. 1).

### Abundance of the TCRβ CDR3 genes transcripts

**Figure 2:**
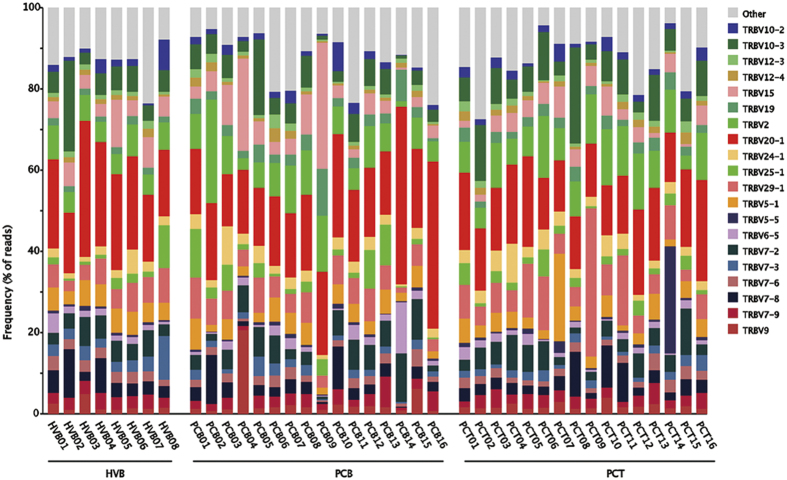
The top-20 expressed gene of the V gene segments in each group. No significant differences were detected between groups. The TRBV20-1 and TRBV2 variants were the most frequent in most samples.

We analyzed the highly expressed V and J gene segments, as well as the V-J gene pair, in all three groups, respectively. The top-20 genes expressed in each sample are listed in Fig. 2. Similar patterns were found in all these samples, and no significant difference was detected among the three groups. In particular, for the V gene, the TRBV20-1 and TRBV2 variants were commonly overexpressed in all samples (Fig. 2), while for the J gene, the TRBJ2-7 and TRBJ2-1 were the most abundant variants (Fig. S1). In addition, the TRBV20-1;TRBJ2-7 and TRBV20-1;TRBJ2-1 were the most highly expressed gene pairs in these samples.

**Figure 3:**
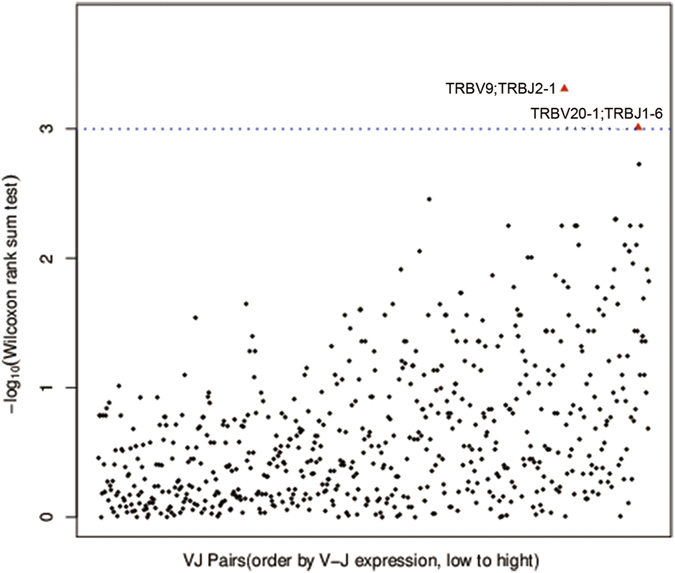
The VJ pairs compared in tumor tissue and blood samples from the 16 patients. Wilcoxon rank sum tests were performed to assess each VJ pair, and P < 0.001 was considered as significant. The TRBV9:TRBJ2-1 and TRBV20-1:TRBJ1-6 were found significantly higher.

To study the relationship between TCR repertoires in the tumor and blood samples in the 16 patients, we performed Wilcoxon rank sum tests to assess the VJ pairs detected. Two VJ pairs (TRBV9:TRBJ2-1 and TRBV20-1:TRBJ1-6) were found with significantly higher levels of expression (Fig. 3). Of note, TRBV20-1 and TRBJ2-1 have been reported as the highest expressed V and J gene variants, respectively, in healthy individuals^13^,^18^.

### Diversities of TCR repertoire in different groups

**Figure 4:**
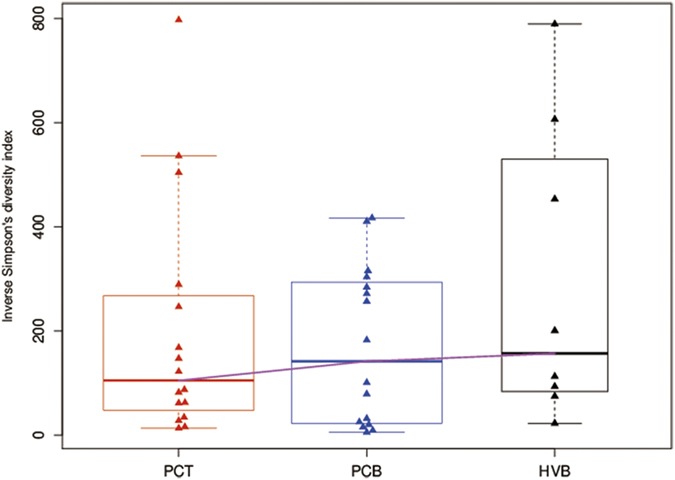
Diversity of TCR repertoires was similar among groups. Inverse Simpson’s diversity index was used to assess the diversity of TCR repertoires, and no significant differences were found among these groups.

**Figure 5:**
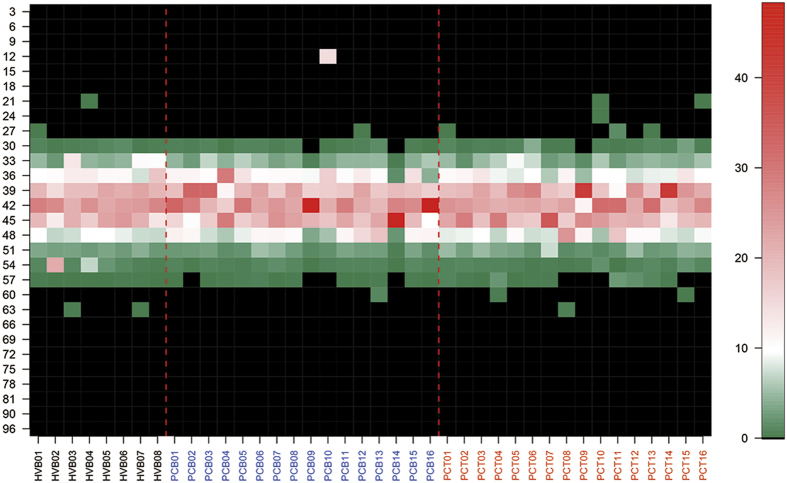
Complementary determining region 3 (CDR3) length usage difference was not detected in groups. The median lengths were 42 base pairs and normal distribution was found in all three groups.

We used the inverse Simpson’s index to measure the diversity levels of the TCR repertoire in different groups, and no significant differences were found among the groups (Fig. 4). Moreover, we assessed the diversity of CDR3 by analyzing the transcript length distribution. The length of CDR3 was mainly between 39–45 base pairs (bp), and there was no significant difference with regards to the length distribution between groups (Fig. 5).

### TCR repertoires overlap within and between samples

**Figure 6:**
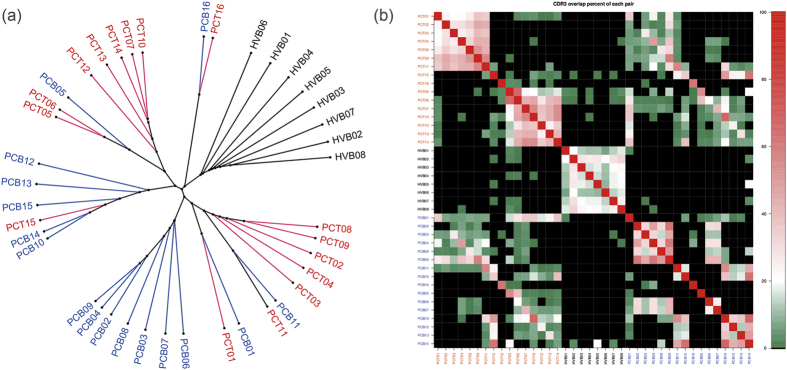
Overlap of TCR repertoires in patients. (**a**) The similarity of TCR repertoires from the 16 patients and 8 healthy controls was analyzed. Healthy individuals formed an independent cluster. Tumor tissue and blood samples from patients were mixed together but some clusters were formed. (**b**) Fussy heat map showed two clusters in tumor tissue and blood samples from the patients.

To compare the similarity of TCR repertoires between tumor tissue and blood samples from patients, and between blood samples from patients and controls, we calculated the rate of overlap (Fig. 6a) and further transformed the data into a fuzzy heat map (Fig. 6b). Two significant clusters could be identified in the PCT group, which suggested a strong degree of similarity of the TCR repertoire in certain patients with pancreatic cancer. The two clusters had similar amounts of TILs (1.8 × 10^7^ ± 1.1 × 10^6^ *vs.* 2.0 × 10^7^ ± 5.9 × 10^6^); however, one cluster had significantly higher diversity of TILs than the other (8.1 × 10^4^ ± 1.8 × 10^4^ *vs.* 1.1 × 10^5^ ± 2.9 × 10^4^, P < 0.05; Fig. S2). No other demographic or clinicopathologic parameters such as lymph node metastasis and fibrotic response were found statistically different between the two clusters. Meanwhile, a weaker degree of similarity could also be found in the PCB group. Importantly, both the PCT and PCB groups showed little overlap with the results from the HVB group, which implied that the immune status of patients with pancreatic cancer had been changed.

To further explore the potential common CDR3 in PCT group, we performed a phylogenetic relationship analysis in all CDR3 transcripts that contributed to the overlap between samples, but no significant clusters were determined among these CDR3 regions (Fig. S3). However, we screened all the sequences and found 15 CDR3 sequences and 30 amino acid sequences were rare in the healthy volunteers but were shared by more than 7 patients (in both tumor tissue and blood samples; Table S2). Notably, gene expression products of all the 15 CDR3 sequences were included in the 30 amino acid sequences identified (Table S2).

### Different clonotype abundance ratios of CDR3

**Figure 7:**
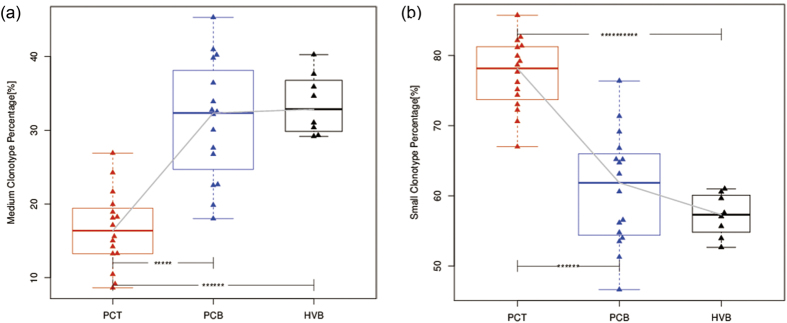
Differences in different clonotypes in the three groups. T cell clonotypes were categorized by different frequencies. (**a**) Tumor tissue samples showed significantly lower levels of medium frequency (0.001%–0.01%) clonotype compared to blood samples from the patients and healthy controls. (**b**) Tumor tissue samples showed significantly lower levels of medium frequency (<0.001%) clonotype compared to blood samples from the patients and healthy controls. ****P < 0.0001, *****P < 10^−5^, **********P < 10^−10^.

CDR3 clonotypes were further classified into five groups according to their abundance, and divided by 0.001%, 0.01%, or 1% of 10^6^ analyzed T cells (Table 1). The small and medium groups occupied between 80% and 90% of clonotypes in the PCT samples. The hyper-expanded and large groups of clonotypes showed insignificant differences between groups (Fig. S4). However, there was a lower proportion of the medium clonotypes and a higher proportion of the small clonotypes in the PCT group compared to those in the PCB and HVB groups (Fig. 7).

## Discussion

Tumor heterogeneity is one the primary obstacles of treatment, and is responsible for drug resistance and the recurrence of solid cancers, including pancreatic cancer^19^,^20^. Theoretically, the clonal evolution model assumes a branched evolution of cancer and provides an explanation for the generation of heterogeneity^21^. Previous reports have confirmed a high level of heterogeneity within a tumor^22^. Recently, the TIL TCR repertoire has been investigated in many types of solid cancer, and has verified that an astonishing amount of heterogeneity can occur in different regions of one single slide of ovarian cancer^11^,^16^, and renal cell carcinoma^17^. To our knowledge, this is the first report regarding the TCR repertoire in pancreatic cancer.

Two VJ pairs were underlined by comparing TCR repertoires between tumor and blood samples; however, some of their components are frequently found in healthy individuals. This finding indicated the effect of common infections, such as Epstein-Barr virus. Given the poor prognosis of pancreatic cancer, the high frequency of these V and J segments found in our study (Fig. 2) further suggests that they are unlikely to be associated with the specific antigens shared between healthy individuals and patients with pancreatic cancer.

Although we have not identified certain subclones of T cells that have specifically expanded and shared in all pancreatic cancer patients, we indeed found a similarity in patients compared to controls. However, the relatively higher similarity determined in both the tumors and blood samples of patients compared to the healthy controls suggested that some subclones commonly exist in pancreatic cancer. These subclones might target the tumor-associated antigens. Given the finding that there was no difference in the subclones with higher levels of abundance, these subclones were probably less abundant. The inefficiency of expansion of some of these subclones might be a result of tumor-induced immune suppression, and could partially explain why pancreatic cancer escapes elimination by the immune system. In addition, a greater degree of overlap was found in the TIL samples than in the blood, which suggested that there were true differences between the TCR repertoires of TILs and circulating T cells. Such differences have already been determined in various other types of cancer^5^,^23^. Our results confirmed the distinctiveness of TILs in pancreatic cancer compared to circulating T cells, and indicates that specific antigens with pancreatic tumors are encountered by the immune system.

In the current limited dataset, we failed to identify specific CDR3 transcripts with regards to the potential homogeneity of pancreatic cancer. This finding may have been partially due to the high level of heterogeneity of this type of cancer, as well as the diversity of the basic congenital TCR repertoires among individuals. Another reason is that T cells, unlike B cells that consistently enhance antibody affinities by evolving, undergo clonal selection and require antigen-presenting cells to provide processed antigens^24^. This makes the study of T cells more difficult. Efficient approaches are currently unavailable, which limits the functional analysis of the CDR3 regions.

However, given the heterogeneity of TCR repertoires among different individuals, we identified some CDR3 and amino acid sequences shared in some patients. This finding could still be meaningful since it seemed hopeless to identify the common TCR in all patients. The 15 CDR3 sequences and corresponding amino acid sequences also suggested a subgroup of patients from a molecular point of view, although we did not find demographic or clinicopathological characteristics these patients shared in common when the patients were grouped by certain sequence. It could be valuable to make an in-depth analysis of these identified TCR gene and amino acid sequences since some of them were associated with tumor-associating antigens and might be helpful to control tumors. For instance, mesothelin was found with high expression in pancreatic cancer and listeria monocytogenes-expressing mesothelin boost vaccine has been recently reported to increase clinical response and survival in pancreatic patients^25^. Other tumor-associated antigens like carbohydrate antigen 19-9 was also used as a target for engineered T cells^26^. Thus, further study on these sequences using TCR T cells or chimeric antigen receptor T cells may be valuable to test their anti-tumor effects.

Mutations accumulated as the tumor evolves. Low-frequency subclones may regulate the development of the dominant subclone within cancer^27^. Although no evidence showed the correlation between the frequency of cancer cell subclones and the frequency of according T cell subclones, the frequency distribution of T cell clonotypes may implicate the complexity of heterogeneity within the tumor. With regards to the different sizes of clonotypes, we did not analyze the rare ones as they were quite infrequent and our method was insufficient to ascertain the results from single cells. Although most unselected T cell subclones were found at a low level, the impressive degree of overlap particularly in the PCT group in particular suggested that some of the subclones were specific to pancreatic cancer. On the other hand, the clonotypes with increased levels of frequency reflected the large degree of immune stress within tumors, which could manifest as increased numbers of novel antigens that resulted from genetic mutations within cancer cells. Additionally, the reduced frequency of clonotypes with a median size may result from a seesaw effect with small clonotypes. Besides, the significantly increased median size might be indicative of T regulatory cells within tumors.

In general, there was no significant difference between patients with pancreatic cancer and healthy volunteers with regards to their TCR repertoires. No differences were found either between the samples of tumor tissue and the blood samples from patients. However, some unidentified CDR3 regions of T cells were indeed associated with pancreatic cancer. Unfortunately no relevant databases exist currently to assess these. We also confirmed the heterogeneity of pancreatic cancer, and through the characteristic distribution of clonotypes, we hypothesize that the cancer cells undergo immunological adaption and therefore escape from local immune surveillance.

## Methods

### Patients and sample collection

This study enrolled 16 patients with pancreatic ductal adenocarcinoma and 8 healthy volunteers as controls. All patients had been diagnosed by tissue pathology, and all had undergone surgery at the Second Affiliated Hospital, Zhejiang University School of Medicine between July 2012 and June 2013. None of these patients had undergone preoperative chemotherapy or radiotherapy. The basic demographic information of the patients is shown in Table 2.

Tumor tissue samples were placed in liquid nitrogen within 30 min after excision, and stored at −80 °C until analysis. The matched peripheral blood samples of the patients were collected into K2EDTA tubes before surgery, and centrifuged at 1,300 g for 15 min at 4 °C within 16 hours after surgery. DNA was extracted from peripheral blood mononuclear cells, which were isolated using lymphocyte separation medium (Beyotime Biotechnology, Shanghai, China). The peripheral blood samples of healthy volunteers were collected in the same method as described above. All samples were classified into three groups: HCT, HCB, and HVB. The protocol of this study was in accordance with the Declaration of Helsinki, and was approved by the Human Ethic Committee of the Second Affiliated Hospital, Zhejiang University School of Medicine. Written informed consent was acquired from all patients and volunteers.

### Multiple-PCR and high-throughput sequencing

We used multiple-polymerase chain reaction (PCR) and 5′ rapid-amplification of cDNA ends (RACE) to capture CDR3 regions using a specifically designed set of primers for the TCRβ CDR3 V and J regions (BGI, Shenzhen, China; Table S3). The captured regions were then sequenced on high-throughput sequencing platform (Hiseq2000; Illumina, San Diego, CA, USA). Using the alignment tool (BGI), we filtered the sequencing background and aligned the raw data to V and J gene segments in the international ImMunoGeneTics (IMGT^®^) database^28^. Several methods were used to eliminate PCR and sequencing errors. (1) Aquirement of reads with appropriate paired-ends (PE): reads without ends or with ends shorter than 50 bp were considered appropriate, with PE1 ≥ 60 bp and PE2 ≥ 50 bp. (2) Elinimate low-quality reads: the proportion of unidentified bases in the sequence should be less than 5%, and the average base quality should be never lower than 15. Bases at the end of reads with base quality less than 10 were deleted. (3) Merge paired-ends into contigs: PE1 and PE2 of reads with paired-ends should have an overlap of 10 bp or more, and the mismatch in the overlap region should be less than 2.

### CDR3 sequence analysis

We analyzed the V and J gene frequency, frequency distribution of clonotypes, and the number of total peptide sequences for CDR3, in addition to the total sequence numbers of the V and J gene segments in each sample. To assess the similarity and differences in the TCRβ repertoires among the three groups of samples, we calculated the inverse Simpson’s index of diversity, the metric overlap of TCRβ CDR3, and the abundance ratios of different clonotypes^29^.

### Screen of cancer specific TCR gene and amino acid sequences

We defined a sequence found in more than 7 samples in both PCT and PCB groups and less than 3 samples in HVB group as a candidate for pancreatic cancer specific TCR gene or amino acid sequence. Expression of the certain sequence was calculated as the ratio of reads of the certain sequence and the total reads.

### Statistical analysis

Diversity levels and differences among groups were analyzed by one-way ANOVA, and P-values less than 0.05 were considered as statistically significant. For VJ pair comparison between tumors and blood samples of patients, Wilcoxon rank sum tests were performed and P-values less than 0.001 were considered as statistically significant.

## Additional Information

**How to cite this article**: Bai, X. *et al.* Characteristics of Tumor Infiltrating Lymphocyte and Circulating Lymphocyte Repertoires in Pancreatic Cancer by the Sequencing of T Cell Receptors. *Sci. Rep.* **5**, 13664; doi: 10.1038/srep13664 (2015).

## Supplementary Material

Supplementary Information

## Figures and Tables

**Table 1:** Definitions of clonotype groups classified according to frequency.

Clone type	Clone size
Rare	Single-cell events
Small	More than single event, but less than 0.001%
Medium	0.001%–0.01%
Large	0.01%–1%
Hyperexpanded	>1%

**Table 2:** Demographic data for the patients in this study.

Variable	Patients (n = 16)
Sex	
Male: female	11: 5
Age, median (range), year	63 (39–78)
Tumor stage	
I: II: III: IV	1: 10: 3: 2
Lymph node involvement	
Positive: negative unknown	7: 8: 1
