# Supplementary material for: Characteristics of Tumor Infiltrating Lymphocyte and Circulating Lymphocyte Repertoires in Pancreatic Cancer by the Sequencing of T Cell Receptors
Source: Sci Rep. 2015 Sep 2;5:13664. doi: 10.1038/srep13664 (PMC4556988; doi:10.1038/srep13664)
Supplement: Supplementary Information [file srep13664-s1.doc]

**Characteristics of Tumor Infiltrating Lymphocyte and Circulating Lymphocyte Repertoires in Pancreatic Cancer by the Sequencing of T Cell Receptors**

Xueli Bai^1,2,†^, Qi Zhang^1,2,†^, Song Wu^3,4,†^, Xiaoyu Zhang^1^, Mingbang Wang^5^, Fusheng He^5^, Tao Wei^1^, Jiaqi Yang^1^, Yu Lou^1^, Zhiming Cai^3^, Tingbo Liang^1,2,6,*^

^1^ Department of Hepatobiliary and Pancreatic Surgery, the Second Affiliated Hospital, Zhejiang University School of Medicine, Hangzhou, China

^2^ Key Laboratory of Cancer Prevention and Intervention, the Second Affiliated Hospital, Zhejiang University School of Medicine, Hangzhou, China

^3^ National-regional Key Technology Engineering Laboratory for Clinical Application of Cancer Genomics, Shenzhen Second People’s Hospital, the First Affiliated Hospital of Shenzhen University, Shenzhen, China

^4^ Siteman Cancer Center, Washington University in St. Louis, St. Louis, Missouri, USA

^5^ BGI-tech, BGI-Shenzhen, Shenzhen, China

^6^ Zhejiang University; Collaborative Innovation Center for Cancer Medicine, Guangzhou, China

^†^ These authors contributed equally to this work.

^*^ Corresponding author. Address: Department of Hepatobiliary and Pancreatic Surgery, the Second Affiliated Hospital, Zhejiang University School of Medicine, No. 88 Jiefang Road, Hangzhou 310009, China. Tel & Fax: +86 571 87315005. Email: liangtingbo@zju.edu.cn

**Supplementary tables**

**Table S1 The numbers of sequencing reads and unique peptides from each individual.**

| **ID** | **PCT** | | **PCB** | | **HVB** | |
| --- | --- | --- | --- | --- | --- | --- |
|  | **TCR CDR3** | | **TCR CDR3** | | **TCR CDR3** | |
|  | **Clonotype** | **Unique peptides** | **Clonotype** | **Unique peptides** | **Clonotype** | **Unique peptides** |
| Patient 1 | 96,052 | 65,923 | 84,372 | 73,270 | - | - |
| Patient 2 | 93,182 | 66,328 | 89,010 | 76,767 | - | - |
| Patient 3 | 108,587 | 74,630 | 91,277 | 76,013 | - | - |
| Patient 4 | 76,287 | 54,811 | 37,167 | 28,195 | - | - |
| Patient 5 | 168,913 | 129,989 | 98,544 | 86,677 | - | - |
| Patient 6 | 86,711 | 66,888 | 75,471 | 54,095 | - | - |
| Patient 7 | 85,873 | 65,306 | 32,815 | 23,886 | - | - |
| Patient 8 | 55,693 | 39,237 | 138,045 | 124,657 | - | - |
| Patient 9 | 73,890 | 51,276 | 38,190 | 28,481 | - | - |
| Patient 10 | 134,008 | 114,790 | 85,733 | 74,388 | - | - |
| Patient 11 | 63,359 | 46,468 | 74,467 | 57,145 | - | - |
| Patient 12 | 119,197 | 83,563 | 80,830 | 72,097 | - | - |
| Patient 13 | 83,901 | 59,372 | 54,165 | 42,633 | - | - |
| Patient 14 | 107,735 | 88,074 | 26,533 | 18,196 | - | - |
| Patient 15 | 32,827 | 21,134 | 143,843 | 107,463 | - | - |
| Patient 16 | 116,140 | 83,708 | 67,031 | 50,714 | - | - |
| Volunteer 1 | - | - | - | - | 106,686 | 76,074 |
| Volunteer 2 | - | - | - | - | 80,976 | 61,448 |
| Volunteer 3 | - | - | - | - | 101,132 | 77,753 |
| Volunteer 4 | - | - | - | - | 99,921 | 73,725 |
| Volunteer 5 | - | - | - | - | 96,244 | 77,577 |
| Volunteer 6 | - | - | - | - | 94,829 | 69,304 |
| Volunteer 7 | - | - | - | - | 85,884 | 63,792 |
| Volunteer 8 | - | - | - | - | 82,545 | 62,450 |

**Table S2 Shared CDR3 and amino acid** sequences in some pancreatic cancers.

| **CDR3 sequence** | **Amino acid sequence** |
| --- | --- |
| TGCAGCGTTGACGAAACAGGGGATACTGAAGCTTTCTTT | CSVDETGDTEAFF |
| TGCAGTGCCACGACTAGCGGGAGGGACACGCAGTATTTT | CSATTSGRDTQYF |
| TGCAGTGCTAGAGATATCGGGGGTATTCAATACGAGCAGTACTTC | CSARDIGGIQYEQYF |
| TGCAGTGCTAGAGATGGGGATGGTGCGCTCTACGAGCAGTACTTC | CSARDGDGALYEQYF |
| TGCAGTGCTAGCCCCCAGTCCTACGAGCAGTACTTC | CSASPQSYEQYF |
| TGCGCCAGCAGTGACGGGGCGGCGGGGCTTGAAGCTTTCTTT | CASSDGAAGLEAFF |
| TGTGCCACCAGCAGAGATGCCGGGGCTAGGGGCTACACCTTC | CATSRDAGARGYTF |
| TGTGCCACCAGCAGAGATGGGACAGACACTGAAGCTTTCTTT | CATSRDGTDTEAFF |
| TGTGCCACCAGTGACGCGGACATTGGCATCTATGGCTACACCTTC | CATSDADIGIYGYTF |
| TGTGCCAGCAGCCGACCGGGCTACGAGCAGTACTTC | CASSRPGYEQYF |
| TGTGCCAGCAGCTCCGGACTAGCGGGGGTTGAGCAGTTCTTC | CASSSGLAGVEQFF |
| TGTGCCAGCAGCTTGGTTCCGAATAGCAATCAGCCCCAGCATTTT | CASSLVPNSNQPQHF |
| TGTGCCAGCAGTGAAACCACTAGCGGGAGGACCCAGTACTTC | CASSETTSGRTQYF |
| TGTGCCAGCAGTGAAGTGGGGTCGGGCAATCAGCCCCAGCATTTT | CASSEVGSGNQPQHF |
| TGTGCCAGCAGTGAGCAGTTATACGAGCAGTACTTC | CASSEQLYEQYF |
| - | CASSL |
| - | CASSLADTQYF |
| - | CASSLEASGGYEQYF |
| - | CASSLGGGETQYF |
| - | CASSLGGSSYNEQFF |
| - | CASSLGGTDTQYF |
| - | CASSLGGYGYTF |
| - | CASSLGSEQYF |
| - | CASSLGSSYEQYF |
| - | CASSLTDTQYF |
| - | CASSPGDEQFF |
| - | CASSPQGYEQYF |
| - | CASSQDSYEQYF |
| - | CASSQGETQYF |
| - | CASTGGYGYTF |

**Table S3 The primers used in the multiple polymerase chain reaction.**

| **Primer** | **Sequence** |
| --- | --- |
| TRBV2TRBV2F | ATTTCACTCTGAAGATCCGGTCCAC |
| TRBV3-1TRBV3-1-F4 | AAACAGTTCCAAATCGMTTCTCAC |
| TRBV4-1TRBV4-1/2/3-F4 | CAAGTCGCTTCTCACCTGAATG |
| TRBV5-4TRBV5-4/5/6/8-F4 | TCAGGTCGCCAGTTCCCTAAYTAT |
| TRBV6-4.1TRBV6-4.1-F | CACGTTGGCGTCTGCTGTACCCT |
| TRBV6-8TRBV6-8/5/1.2-F | CAGGCTGGTGTCGGCTGCTCCCT |
| TRBV6-9TRBV6-9/7/1.1/6-F | CAGGCTGGAGTCAGCTGCTCCCT |
| TRBV6-4.2TRBV6-4.2-F | AGTCGCTTGCTGTACCCTCTCAG |
| TRBV6-2TRRBV6-2/3-F | GGGGTTGGAGTCGGCTGCTCCCT |
| TRBV7-2TRBV7-2/4/6/7/8-F4 | GGGATCCGTCTCCACTCTGAMGAT |
| TRBV7-3TRBV7-3-F4 | GGGATCCGTCTCTACTCTGAAGAT |
| TRBV7-9TRBV7-9-F4 | GGGATCTTTCTCCACCTTGGAGAT |
| TRBV9TRBV9F | CCTGACTTGCACTCTGAACTAAACCT |
| TRBV10-1TRBV10-1-F4 | CCTCACTCTGGAGTCTGCTGCC |
| TRBV10-2TRBV10-2/3-F4 | CCTCACTCTGGAGTCMGCTACC |
| TRBV11-1TRBV11-1/2/3-F4 | GCAGAGAGGCTCAAAGGAGTAGACT |
| TRBV12-3.2TRBV12-3.2/5.2 | GAAGGTGCAGCCTGCAGAACCCAG |
| TRBV12-3.1TRBV12-3.1/4/5.1-F | GAAGATCCAGCCCTCAGAACCCAG |
| TRBV13TRBV13-F4 | TCGATTCTCAGCTCAACAGTTC |
| TRBV14TRBV14F | GGAGGGACGTATTCTACTCTGAAGG |
| TRBV15TRBV15F | TTCTTGACATCCGCTCACCAGG |
| TRBV16TRBV16-F2 | CTGTAGCCTTGAGATCCAGGCTACGA |
| TRBV18TRBV18-F4 | TAGATGAGTCAGGAATGCCAAAG |
| TRBV19TRBV19F | TCCTTTCCTCTCACTGTGACATCGG |
| TRBV20-1TRBV20-1-F4 | AACCATGCAAGCCTGACCTT |
| TRBV24-1TRBV24-1-F2 | CTCCCTGTCCCTAGAGTCTGCCAT |
| TRBV25-1TRBV25-1F | GCCCTCACATACCTCTCAGTACCTC |
| TRBV27-1TRBV27-1 | GATCCTGGAGTCGCCCAGC |
| TRBV28TRBV28 | ATTCTGGAGTCCGCCAGC |
| TRBV29-1TRBV29-1-F4 | AACTCTGACTGTGAGCAACATGAG |
| TRBV30TRBV30-F5 | CAGATCAGCTCTGAGGTGCCCCA |
| TRBJ1-1TRBJ1.1-R2 | CTTACCTACAACTGTGAGTCTGGTG |
| TRBJ1-2TRBJ1.2R | CTTACCTACAACGGTTAACCTGGTC |
| TRBJ1-3TRBJ1.3R | CTTACCTACAACAGTGAGCCAACTT |
| TRBJ1-4TRBJ1-4 | AAGACAGAGAGCTGGGTTCCACT |
| TRBJ1-5TRBJ1.5R | CTTACCTAGGATGGAGAGTCGAGTC |
| TRBJ1-6TRBJ1.6R | CATACCTGTCACAGTGAGCCTG |
| TRBJ2-1TRBJ2.1R | CCTTCTTACCTAGCACGGTGA |
| TRBJ2-2TRBJ2.2R | CTTACCCAGTACGGTCAGCCT |
| TRBJ2-3TRBJ2.3R | CCGCTTACCGAGCACTGTCAG |
| TRBJ2-4RBJ2-4 | AGCACTGAGAGCCGGGTCC |
| TRBJ2-5TRBJ2.5-R2 | CGAGCACCAGGAGCCGCGT |
| TRBJ2-6TRBJ2.6R | CTCGCCCAGCACGGTCAGCCT |
| TRBJ2-7TRBJ2.7-R2 | CTTACCTGTGACCGTGAGCCTG |

Base Y refers to C/T, and base M refers to A/C.

**Supplementary figures**

**
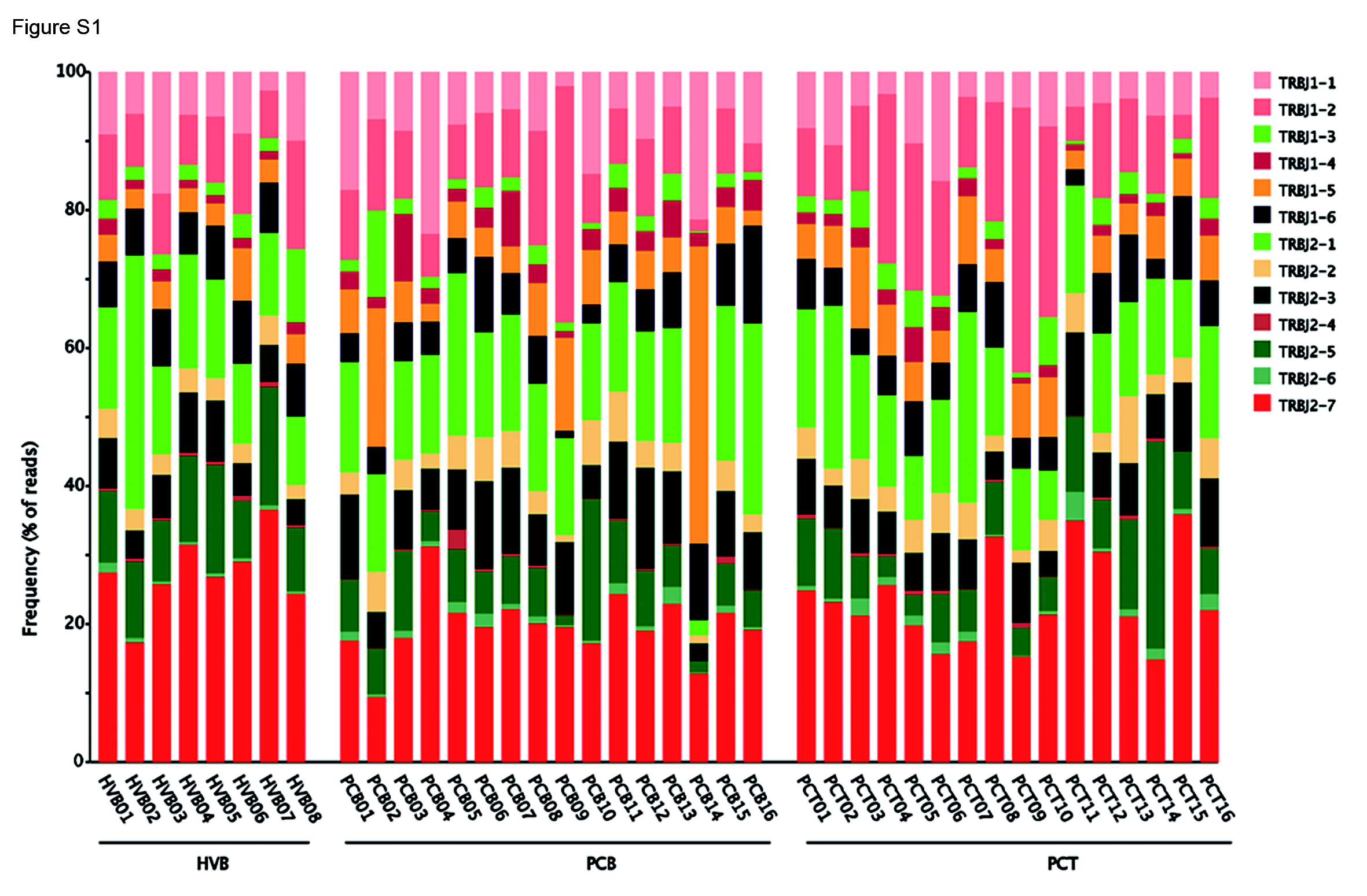
**

**Figure S1 The top-20 expressed gene of the J gene segments in each group.** No significant differences were detected between groups. The TRBJ2-7 and TRBV2-1 variants were the most frequent in most samples.


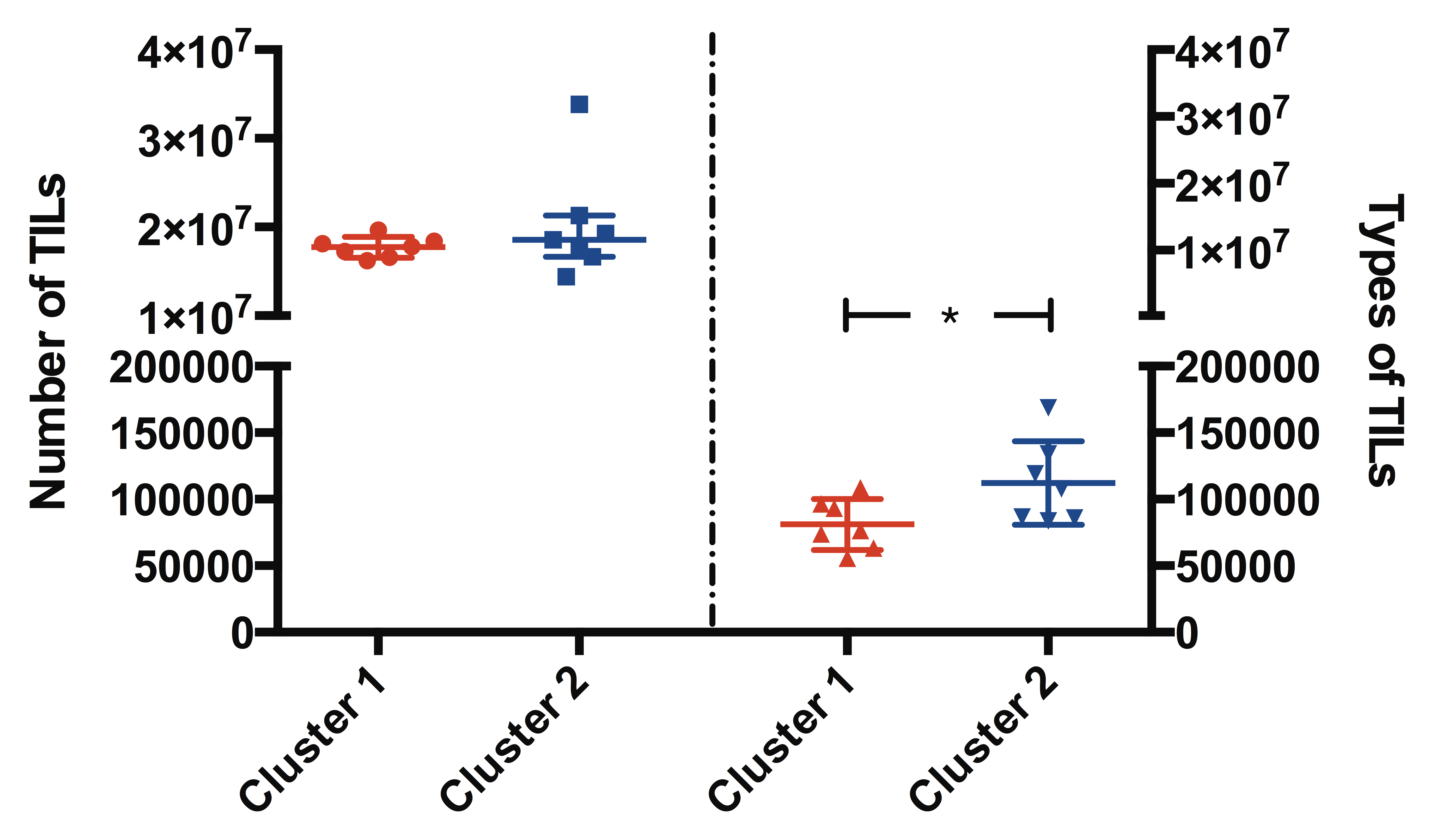


**Figure S2 Number and type of tumor infiltrating lymphocytes (TILs) in the two clusters identified by overlap of T cell receptor repertoires.** The number and type of the TILs were required from the original sequencing data. Cluster 1 was composed of PCT01, PCT02, PCT03, PCT04, PCT08, PCT09, and PCT11. Cluster 2 included PCT05, PCT06, PCT07, PCT10, PCT12, PCT13, and PCT14. The total numbers of TILs were similar between both clusters. However, cluster 2 had more types of TILs compared to cluster 1. * P < 0.05.

**
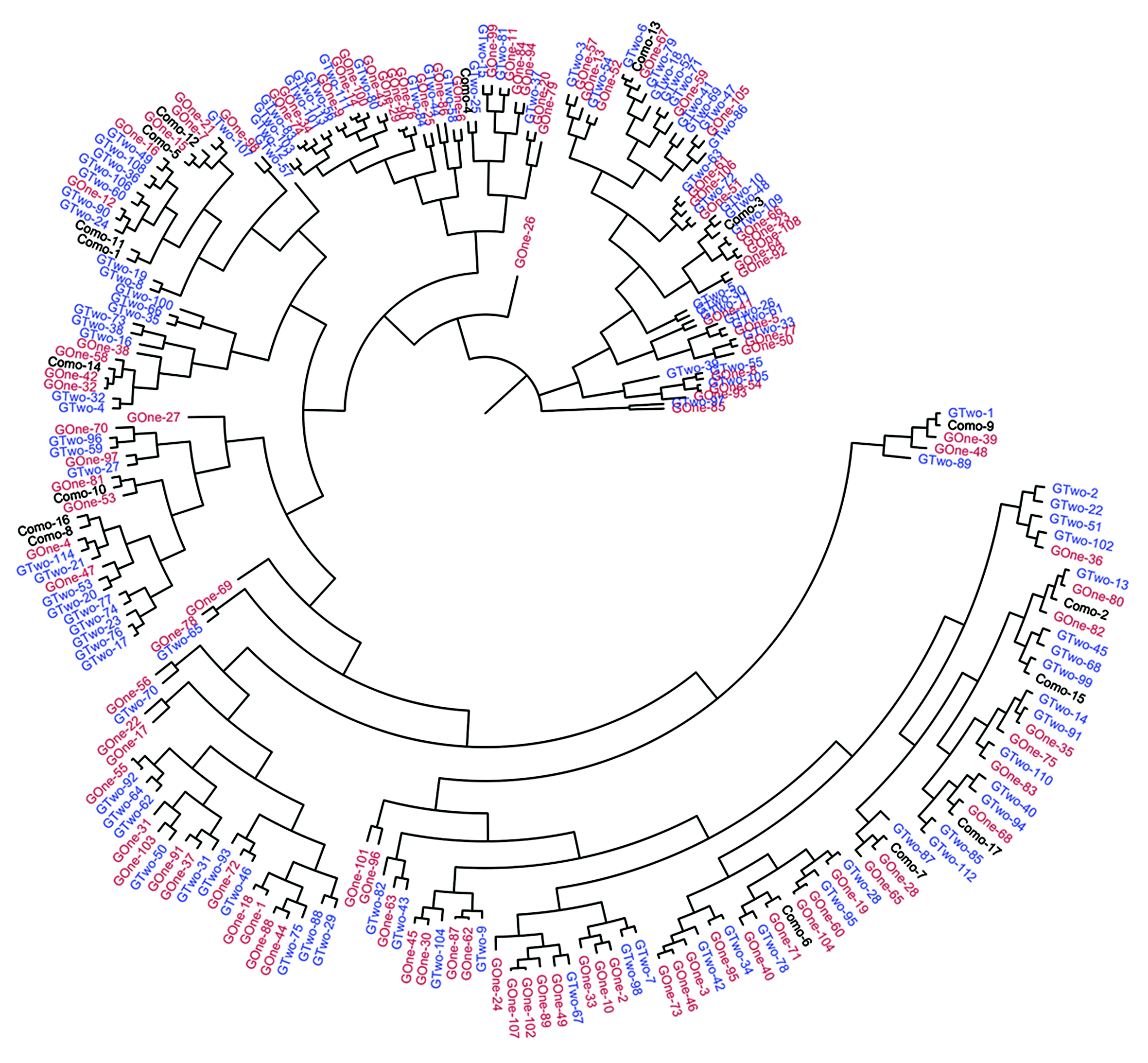
**

**Figure S3 The relationship of CDR3 transcripts in all samples.** All CDR3 transcripts beginning with GOne (Red) indicate those contribute to the first cluster of PCT group in Figure 6B. Similarly, all CDR3 transcripts beginning with GTwo (blue) indicate those contribute to the second cluster of PCT group, and all CDR3 transcripts beginning with Como (black) indicate those contribute to both clusters of PCT group in Figure 6B. It fails to differentiate GOne and GTwo transcripts as two clusters here.

**
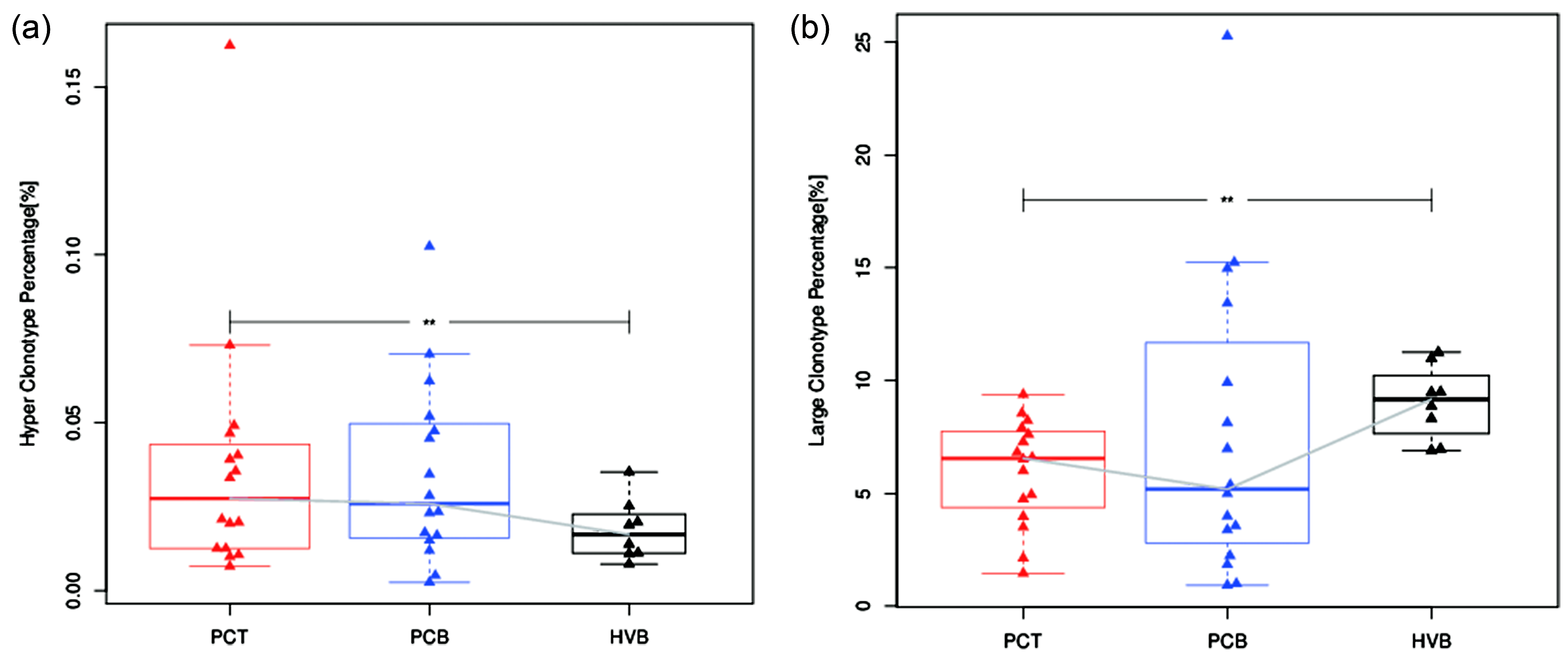
**

**Figure S4 Differences in** **different clonotypes in the three groups.** T cell clonotypes were categorized by different frequencies. (a) Tumor tissue samples from patients had a higher level of hyperexpanded (> 1%) clonotype of T cells compared to that of blood samples from healthy controls. (b) Tumor tissue samples from patients had a higher level of large frequency (0.01% - 1%) clonotype of T cells compared to that of blood samples from healthy controls. ** P < 0.01.
